# Labor force participation, unemployment and occupational attainment among immigrants in West European countries

**DOI:** 10.1371/journal.pone.0176856

**Published:** 2017-05-05

**Authors:** Anastasia Gorodzeisky, Moshe Semyonov

**Affiliations:** 1 Department of Sociology and Anthropology, Tel Aviv University, Tel Aviv, Israel; 2 Department of Sociology, University of Illinois at Chicago, Chicago, Illinois, United States of America; Queensland University of Technology, AUSTRALIA

## Abstract

The present paper examines modes of immigrants' labor market incorporation into European societies with specific emphasis on the role played by immigrant status (i.e. first-generation immigrants, immigrant descendants and native born without migrant background), region of origin, and gender. The data were obtained from the European Union Labour Forces Survey 2008 Ad-Hoc Module for France, Belgium, UK and Sweden. In order to supplement the results from the country-specific analysis, we replicated the analysis using pooled data from the five rounds of the European Social Survey conducted between 2002 and 2010, for nine 'old immigration' Western European countries together. The analysis centered on two aspects of incorporation: labor force status and occupation. Multinominal, binary logistic as well as linear probability regression models were estimated. The findings suggest that in all countries non-European origin is associated with greater disadvantage in finding employment not only among first-generation immigrants, but also among sons and daughters of immigrants (i.e. second-generation). Moreover, the relative employment disadvantage among immigrant men of non-European origin is especially pronounced in the second-generation. The likelihood of attaining a high-status job is influenced mostly by immigrant status, regardless of region of origin and gender. The results of the study reveal that patterns of labor force incorporation vary considerably across origin groups and across generations. The patterns do not vary as much across countries, despite cross-country differences in welfare state regimes, migration integration policy and composition of migration flows.

## Introduction

The flow of migrants to Western European countries in the post-WWII era is typically explained by an increase in demand for workers in the host countries on the one hand, and by a large labor force supply in countries of origin on the other hand. This increased demand for workers in Western Europe is attributed to rapid economic growth, rising educational levels and declining fertility, coupled with the reluctance of the local population to take low-status, low-paying, menial jobs in the domestic labor market. The demand for workers (mostly for cheap labor) was met by the supply of immigrants and labor migrants from poor, less-developed countries in Asia, North Africa, Sub-Saharan Africa, the Middle East and Latin America, as well as from Eastern Europe [1], [2], [3], [4]. Since the mid-1980s, Western European countries have also experienced significant increases in the flow of asylum seekers and refugee migration, as well as undocumented and 'illegal' migration, primarily from the Middle East, Africa and the constituent republics of the former Yugoslavia [5]. Consequently, the relative size of the foreign-born population in Western Europe has grown steadily, ranging from 9 to 16 percent across most countries at the end of the first decade of the 21st century [6].

Incorporation of immigrants into society has not only become one of the most frequently discussed and debated issues in Europe, but also a major research topic for social scientists. The present paper joins the growing literature on the topic by focusing on modes of immigrants' incorporation into the labor market, with specific emphasis on the role played by generation (immigrants and immigrant descendants), region of origin, and gender on two aspects of labor market outcomes: labor force status and occupational attainment. The first pertains to the likelihood of being employed, unemployed or out of the labor force; the second pertains to the likelihood of employment in a high-status (professional, technician and managerial) job. The two aspects are interdependent. Joining the economically active labor force is a necessary condition for the attainment of an occupational position.

To do so, we analyzed data obtained from the European Union Labour Forces Survey (EULFS) 2008 Ad-Hoc Module: “Labour market situation of migrants and their immediate descendants,” for France, Belgium, the United Kingdom and Sweden. The four countries are characterized by different welfare state regimes and migration policies, thus allowing for the comparison of modes of immigrant labor market incorporation across different social and political contexts in 'old immigration' Western Europe. In order to supplement the results from the country-specific analysis, we replicated the analysis using pooled data from the five rounds of the European Social Survey conducted between 2002 and 2010, for nine 'old immigration' Western European countries together (namely, Belgium, Switzerland, Germany, Denmark, France, the United Kingdom, Netherlands, Norway, and Sweden). The term 'old immigration' countries pertains to the Western European countries where massive flows of international migrants had begun shortly after WWII (most of the immigrants were actually recruited as labor migrants to solve labor shortage problems and to prompt economic development). The 'old immigration' countries have been dealing with issues related to social and economic incorporation of immigrants and their descendants for several decades.

## Theory and setting

### Previous studies

The research on economic integration of immigrants in European societies can be divided into two groups of studies. The first group concentrates on incorporation of immigrants into a single national labor market (for example, [7] for Sweden; [8] for the UK; [9] for Germany; [10] for Belgium). The second group of studies, which is quite small, is composed of studies that examine the integration of immigrants into the labor market of the host country within a cross-national comparative framework. Several comparative studies have focused on disparities between first-generation immigrants and native-born populations in labor force participation and employment rates (e.g. [11], [12], [13]), and earnings (e.g. [14]). These studies reveal that when compared to the native-born population, first-generation immigrants–especially those from non-EU countries–are less likely to become economically active, and are at a disadvantage in the attainment of economic outcomes.

Other comparative studies focused on differences between immigrant groups from various origins and across multiple destinations, but without comparing the outcomes of these groups against those of the native-born populations [15], [16], [17]. One of the main findings emerging from these studies is that first-generation immigrants from predominantly Christian countries are at an advantage in the quest to become active in the labor markets of host Western countries, as compared to other immigrants [15]. Immigrants from non-Christian countries, however, tend to have higher odds for self-employment [16]. Fleischmann and Dronkers [17] revealed that immigrants (and their descendants) from Islamic countries have higher rates of unemployment, while those originating from Western Europe are less likely to be unemployed in comparison to immigrants from other countries.

Heath and Cheung [18] focused on the integration of second-generation from a variety of countries of origin as compared against that of native-born Europeans (without migrant background). Their analysis reveals that for most groups, the offspring of immigrants are less able to avoid unemployment, and are less likely to secure salaried jobs.

Despite the contributions of previous studies to the topic, there is hardly any comparative research with a focus on the economic integration of both first- and second-generation immigrants from different regions of the world as compared to native-born populations (for one notable exception, see [19]). Thus, the present research seeks to advance knowledge by examining *simultaneously* the differential impact of gender, generation, and region of origin on the integration of immigrants into the labor market of host societies as compared to the native population, in Belgium, France, the UK and Sweden. Specifically, this research investigates whether and to what extent, both first- and second-generation immigrants from different regions of origin differ from natives in their incorporation into the labor market; and, once incorporated, the extent to which they differ from the native-born population in the attainment of high-status jobs. In addition to the four-country comparison, we supplement the findings with data of nine 'old immigration' Western European countries pooled together. By so doing, we will be able to provide a broader comparative perspective on the two aspects of labor market incorporation–labor force status and occupational attainment–than that provided in previous studies.

### Theoretical frameworks

According to the ***classic assimilation model*** (e.g. [20], [21]), newly arrived immigrants experience difficulties in the labor market of the host society. The classic assimilation model attributes this difficulty to immigrants’ limited access to information and social networks; restricted knowledge of the new society’s language and culture; inadequate professional skills; lack of host-country educational credentials; and little or no host labor-market experience. With the passage of time and especially over generations, however, after learning the local language and acquiring the local culture, immigrants tend to experience upward socio-economic mobility and to converge with comparable native-born populations (e.g., [22], [23], [24] for North America).

This classic assimilation model, developed in the context of American society (based on the experience of European immigrants at the turn of the twentieth century) has been reformulated in recent years. It was replaced by alternative theoretical models (i.e. new assimilation theory, segmented assimilation), that underscore the differential patterns of assimilation among members of different ethnic groups in America (e.g., [25], [26]). According to the ***segmented assimilation model***, host societies offer unequal and inequitable distributions of possibilities and opportunities to different ethnic groups. While some groups have access to an abundance of opportunities, others face multiple disadvantages, including discrimination. As a result, whereas some groups may experience inter-generational economic upward mobility (either by assimilating into the mainstream of society or through ethnic cohesion), others (or, at least, a sizeable proportion of them) may integrate into the bottom segments of society [27], [28], [29].

The 'segmented assimilation' model contends that the assimilation experience of contemporary second-generation of non-white origin in America differs from that of white immigrants. The model identifies structural barriers associated with prejudice, negative stereotypes and discrimination that prevent non-white ethnic immigrants from achieving full and successful integration into society. The idea was clearly summarized by Zhou ([29], p.983) as follows: “From this perspective, immigrants and ethnic minorities are constrained by the ethnic hierarchy that systematically limit their access to social resources, such as opportunities for jobs, housing, and education, resulting in persistent racial/ethnic disparities in levels of income, educational attainment, and occupational achievement.”

According to Van Tuburgen et al. [15] ethnic hierarchies play a major role in explaining the underachievement of some immigrant groups of non-European ethnic origins in Western Europe. Ethnic hierarchies are shaped and formed by the social distance between the host society’s native population and the immigrant group. The extent of the social distance between any two groups can be determined by differences and distinctions in culture and physical appearance. The more socialy distant natives feel toward an immigrant group, the more pronounced the prejudice and discrimination toward the group. Hence, socioeconomic disadvantages experienced by an immigrant group are likely to increase with the social distance that natives feel toward the group (e.g. [30], [31], [15]).

### Theoretical expectations

Past research in Western European countries has consistently shown that after arrival, immigrants experience substantial disadvantages in their attempts to join the economically active labor force and in securing high-paying jobs (e.g., [19], [11], [12]). According to the classic assimilation model, economic disadvantages encountered by second-generation immigrants are likely to decline or even diminish, because their offspring do not suffer from the disruptions commonly associated with the migration process, and are able to acquire a domestic education and build up work experience in the host country's labor market [32]. Following the logic embodied in the classic assimilation model, we expect *labor market disadvantages to be most evident among first-generation immigrants*, *but to vanish (or greatly decline) among second-generation*.

Recent research in European societies, however, reveals varying levels of socioeconomic outcomes and differential adaptation processes across origin groups in the second-generation [33], [34], [35], [36]. More specifically, research on immigrant groups demonstrates that in most European countries, ethnic minorities, and especially immigrants from non-European countries, are not only geographically concentrated–often in areas of relatively high social deprivation and scarce labor market opportunities [37], [38]–but also experience difficulties in integrating into mainstream European society and economies [19], [8], [32], [39], [18]. These findings are consistent with the logic of the segmented assimilation model (e.g. [26]), which leads us to the following two expectations. First, *labor market disadvantages can be expected to be more pronounced among immigrants of non-European origin than among immigrants of European origin*. *Second*, *with regards to the second-generation*, *immigrants of non-European origin can be expected to experience disadvantages in the labor market*, *whereas immigrants of European origin can be expected to achieve parity with native-born populations without a migrant background*.

Before proceeding with presentation of the data and findings, we provide a brief description of the social setting of the four countries included in the study.

### The setting: Immigration in Belgium, France, Sweden and UK

The four countries included in the study represent three different welfare state regimes, according to Esping-Anderson's classification [40]. The UK belongs to the liberal cluster; France and Belgium can be viewed as conservative welfare states; and Sweden is a prototype of the socio-democratic welfare cluster. All four countries have received significant immigration flows from south of Europe and the Mediterranean, as well as from African, Caribbean and Asian countries. The foreign workers arrived or were invited to resolve domestic labor shortages during a period of reconstruction and industrial growth [41].

While France and the UK, between the early 1950s and the beginning of the 1970s, drew on their colonial ties to attract immigrants (largely from North Africa, and the Caribbean and Indian subcontinent respectively), Belgium never actively attracted migrants from its former colony (Congo) and protectorates, but rather recruited guest workers mainly from Southern Europe and the Mediterranean area. Moreover, residents of Belgium colonies were never granted Belgian citizenship, while inhabitants of British colonies and Commonwealth states (in the 1950s and 1960s) had the right to settle in the UK and receive British citizenship. Sweden, without a history of colonial dominion, maintained an open immigration policy, and consequently attracted large numbers of immigrants from Southern Europe, the Mediterranean and Finland [4], [42], [43].

France, being the largest immigration country in Europe since 1880, decided to stop the recruitment of a salaried immigrant labor force in 1974. Since then, family reunification, repatriation, and asylum seeking, coupled with undocumented migration, have become the principal pathways for migration to France. However, the shortage of skilled and unskilled workers in some sectors of the French economy by the middle of the first decade of the 21st century led to creation of a list of jobs for which foreign workers could apply without constraints. In 2008, foreign-born residents comprised 11.5 percent of the population of France, with Portuguese, Algerians and Moroccans forming the largest groups of immigrants [44].

Belgium officially stopped immigration for third country nationals in 1973. Since then, family reunification, asylum seeking, and employment in a limited number of professions have become the principal migration channels into Belgium for non-EU migrants [42]. In 2008, about 13.5 percent of the population of Belgium was foreign-born [6]. Currently, the largest foreign (by citizenship) groups in Belgium are Italians, French, Dutch, and Moroccans [42].

In common with France and Belgium, the UK also closed its doors to labor migration in early 1970s. After that, migration to the UK continued mostly via family related immigration and inflows of refugees. However, by the mid-1990s, low unemployment rates and significant shortages in the domestic labor force led, once again, to a more relaxed immigration policy. This change in policy in turn culminated, in 2004, in an open-door policy towards migration from the new Eastern European member states of the EU [45]. By 2008, foreign-born residents comprised about 11 percent of the total population of the UK [6]. The largest foreign-born groups residing currently in the UK are Indian, Pakistani, Polish and Irish [45].

In terms of contemporary migration policy, Sweden is often viewed as an exception. It is considered the European country with the most relaxed migration policy (even as a pro-immigration country). Sweden is a principal destination for asylum seekers (largely from Iraq, Somalia and the Balkans), and explicitly invites new labor migrants. Unlike most European countries, migration policy in Sweden did not become more restrictive during the 2000s. By 2008, foreign-born populations comprised 13.9 percent of the population of Sweden [6] with Finns, Poles and Iraqis the main immigrant groups [43].

The performance of the four countries with regards to immigrant integration policy differs substantially, as implied by the Migrant Integration Policy Index (MIPEX). The MIPEX is evaluated via a measure of policies aimed at integrating third-country migrants into EU member states [46]. In the labor market access area, MIPEX includes policies related to eligibility (e.g., ability to accept any employment, ability to take up self-employment, procedures for recognition of academic qualifications, etc.), labor market integration measures, security of employment, and rights associated with employment. Because policies are measured by the same highest European standards across all member states, it can serve as a 'benchmarking' tool for comparing differential levels of performance-integration of immigrants across countries [46].

When comparing the MIPEX scores (in 2005) with regard to the access afforded immigrants to labor markets, substantial differences across the four countries are evident [46]. Sweden received the highest practice score (100), while France was assigned the lowest score (50). Belgium and the UK's performance with regard to immigrant access to labor market activity fell somewhere in between, with scores of 75 and 60 respectively. Indeed, the four countries differ in many aspects, including welfare state regimes, past and present ties to former colonies, and immigration and migrant integration policies. Thus, in what follows, we examine the similarities and differences in the incorporation of immigrants into labor markets in terms of employment, unemployment and occupational attainment, across the various political and socio-economic contexts represented by the four countries.

## Data and methods

### Data and variables

This study is based on data from Eurostat, EU Labour Force Survey (EULFS) 2008 Ad-Hoc Module: “Labour market situation of migrants and their immediate descendants,” for four countries: France, Belgium, the UK and Sweden (The responsibility for all conclusions drawn from the data lies entirely with the authors). The EU Labour Force Survey (http://ec.europa.eu/eurostat/web/lfs/overview) is the largest European household sample survey, providing quarterly and annual data on labour participation of people aged 15 and over and on persons outside the labour force. The national statistical institutes are responsible for collecting the data from national representative samples and forwarding the results to Eurostat in accordance with the requirements of the regulation. The scheme provides an opportunity to create large harmonised cross-nationally comparable dataset at European level in terms of concepts, definitions and set of characteristics in each country.

The response rate in 2008 for the four countries under study was 74.2 in Belgium, 80.8 in Sweden, 84.3 in France and 68 in UK [47]. For further detailed information on sampling procedure in each country, see [47]. The 2008 EULFS achieved samples (persons 15–74) ranged from 21100 in Belgium to 85600 in UK. One possible serious limitation of such dataset for the present study could be lower response rate among migrants, however, “given the plausible results of important variables in 2008 Ad-Hoc Module, the Labor Force Survey seems to be able to catch migrants and descendants of migrants to a reasonable extent”([48], p.6). The analysis reported here pertains to the population between 20 and 64 years of age.

The EULFS 2008 Ad-Hoc Module data provides the necessary data to construct a series of key variables representing immigration status: first-generation immigrants, offspring of immigrants (hereafter, second-generation) and native-born Europeans, as well as indicators for labor market activity. Specifically, *first-generation immigrants* are those born outside the specific country of residence, and whose parents were born outside the country. *Second-generation* are those born in the specific European country, but both of whose parents were born outside the country. *Native-born* are those born in the specific country, and at least one of whose parents was born in the country (or those born outside the country but holding the specific European country’s citizenship and both of whose parents were born in the country).

The immigrant population can be further divided into two major geo-cultural groups. The geo-cultural division is obtained by distinguishing between two major regions of origin: European and non-European (father of the respondent born in Europe, Australia or Northern America versus non-European). The distinction between the two regions pertains to a variety of characteristics, including ethnicity and religion (with non-whites and non-Christians overrepresented in non-European group). However, for the sake of accuracy, we refer to the geo-cultural distinction as European versus non-European origin, while keeping the overlap between region and ethnicity and religion in mind.

It is important to note that due to the anonymization procedure carried out by Eurostat, Turkey and European countries outside the EU27 and EFTA are merged together and compose one category named 'other Europe'. In the four countries included in the study, the percentage of respondents belonging to the category 'other Europe including Turkey' is very small (about 1% of the general population), and the overwhelming majority of immigrants can be divided between those of European region of origin and those of non-European region of origin. The 'other Europe including Turkey' categories by generation are included in the multivariate analysis only for control purposes.

In addition, a series of socio-demographic variables traditionally used as predictors of labor market activity and labor market outcomes were included in the analysis (albeit mainly for control purposes). They are gender, age (mean value of five years interval), marital status (married = 1), number of children below age 14 (except for Sweden, where the data on number of children are not available), and education as a series of dummy variables: low (low secondary), middle (upper secondary) and high (tertiary) educational level.

The dependent variables pertain to two different aspects of labor market outcomes: *labor force status*, and *occupational status*. *Labor force status* is comprised of three categories: employed (during the reference week), unemployed, and out of the labor force. This variable, consequently, qualitatively distinguishes between three positions in the labor market: those who are able and successful in finding gainful employment (i.e. employed); those who are interested in economic activity and searching for employment, but unable to find employment and secure a job (i.e. unemployed); and those who do not join the economically active labor force for a variety of reasons, including cultural norms and familial constraints (i.e. out of the labor force). *Occupational status* is comprised of a dummy variable that distinguishes between those engaged in high-status lucrative jobs (professional, technician and managerial jobs), and those engaged in other occupations (blue collar, service, sales and clerical jobs).

Complete cases analysis was performed for each dependent variable. For *labor force status* analysis, percent of the missing cases (almost entirely due to lack of information on country of birth of respondent and/or his/her parents) does not exceed 3% except for the UK (16%). The populations with missing data do not differ considerably form those with full information. We believe, therefore, that there is no serious selectivity problem. For *occupation status* analysis, additional missing cases were rather small and only 1% of cases in the Swedish sample, 2.3% in the Belgium and UK samples, and 5% in the French sample were excluded from the analysis.

A supplementary analysis was performed with data obtained from five rounds (2002, 2004, 2006, 2008, and 2010) of the European Social Survey (ESS) for the following nine 'old immigration' countries in Western Europe: Belgium, Switzerland, Germany, Denmark, France, the United Kingdom, Netherlands, Norway, and Sweden [49]. In each country, information was gathered from a representative national sample of the resident populations aged 15 and over. Sample size for each country in every year ranged from 1500 to 3000. For detailed information on ESS methodology, see: http://www.europeansocialsurvey.org/methodology/.

The nine 'old immigration' countries are lumped together into one category, referred to as Western Europe. The variables available in the ESS for the Western Europe category are similar to those included in the country-specific data. The analysis for Western Europe was also conducted on a population group aged between 20 and 64, thus, enabling further comparisons between Western Europe and each of the four countries. A weighting procedure (provided by ESS) was applied, to adjust each country's sample size to its proportion with regards to the overall European population. In order to increase the number of cases (mostly the number of immigrants) and in order to achieve more reliable statistical estimates, all five rounds were pooled into one sample, controlling for year of survey in the analysis. Due to missing information 1.4% cases were excluded from '*labor force status*' analysis and 1.8% were excluded from *occupational status* analysis.

The information on sample size for each country and for the pooled data is displayed in S1 Appendix.

### Methods

To evaluate **employment disadvantage (or advantage)** of different sub-groups of immigrants (as compared to natives), we estimated country-specific multinomial logistic regression equations. Each equation provided estimates of the relative odds of being unemployed and being out of the labor force (versus being employed) for the sub-groups of immigrants (i.e. first-generation European origin immigrants, first-generation non-European origin immigrants, second-generation European origin and second-generation non-European origin) as compared to native Europeans. The equations were estimated while controlling for age, age squared (to control for the curve-linear relationship between employment and age), marital status, and education. Therefore, the coefficients for each sub-group of immigrants for being unemployed/out of the labor force can be viewed as indicators of net advantage (or disadvantage) enjoyed by each group with regards to incorporation into the labor market of the host society.

In light of recent criticism concerning the comparability of logistic regression estimates over groups and across samples [50], we replicated the analysis on sub-samples of persons in the active labor force, utilizing the linear probability model. From the several approaches suggested by Mood [50], we chose the linear probability model as a most appropriate solution for the purpose of presented analysis because it focuses on averaged effects. The estimated coefficients of linear probability models predicting probability of unemployment are presented in S3 Appendix. The results regarding comparison over groups and across country samples obtained by linear probability models resemble the results of logistic models (displayed and discussed in the paper).

In order to evaluate the relative net odds of **attaining high-status jobs** (professional, technician and managerial jobs, PTM) by different sub-groups of immigrants (as compared to natives) we estimated a series of logistic regression equations. In each equation, employment in a high-status occupation was taken as a function of age, age squared (to control for the curve-linear relationship between employment and age), marital status, education, number of children below 14 and a series of dummy variables representing various sub-groups of immigrants: first-generation European origin immigrants, first-generation non-European origin immigrants, second-generation European origin, and second-generation non-European origin (native-born Europeans without migrant background are the omitted category).

When estimating labor market outcomes (i.e. occupational status) across different sub-groups, one should take also into consideration the differential selectivity of sub-groups into the economically active labor force. This phenomenon is especially relevant for women of non-European origin, because some arrived from countries of origin that tend to restrict the participation of women in the public sphere and in economic activities due to traditional values (e.g. [51], [34]). Thus, in order to control for the potential impact of differential selectivity into the labor market on the estimated odds of employment in high-status jobs, we also estimated multinomial logistic regressions with a dependent variable with three categories: high-status occupations; low-status occupations; and not employed (the low-status occupations served for us as the comparison category). This estimation procedure enabled us to predict the odds of employment in high-status jobs versus low-status jobs, while taking into account the differential odds for not being part of the economically active labor force. The findings resulting from the analysis reveal no meaningful differences between the results of the multinomial analysis (controlling for the impact of selectivity into the economically active labor force) and the results of the logistic regression (without such control). Following these findings and for the sake of clarity and parsimony, we decided to present and discuss in the main text the results of the logistic regression. The results of multinomial regressions are presented in the S5 Appendix. In addition, we replicated the analysis using a linear probability model. The results of the linear probability models (presented in the S7 Appendix) regarding comparison over groups and country samples strictly resemble the results of the logistic models discussed here.

After estimation of all models for four separate countries (based on EULFS data), we conducted supplementary models for the pooled data for *nine* '*old immigration*' *Western European countries (*obtained from ESS). The models include a series of dummy variables for each country and a series of dummy variables representing the ESS round. By so doing, we treated the labor markets in the nine 'old immigration' Western European countries as one labor market while controlling for possible variation across countries. However, we also estimated models that did not include a series of dummy variables for each country (without controlling for possible variation across countries), and received results that led to the similar conclusions.

## Results

### Descriptive statistics

Tables 1 and 2 display three categories of position in the labor market for native-born and for the different sub-groups of immigrants for men and women, respectively, in the UK, France, Belgium and Sweden, as well as for Western Europe as a whole. The data clearly reveals that the percentage of unemployed among all sub-groups of immigrant men is higher than that of native-born men in all countries, with only one exception. In the UK, the share of unemployed among immigrants of European origin is somewhat lower than that of the native-born population.

**Table 1:** Immigrants in the labor market of West European countries: Descriptive statistics, men, %. Data Source: EU Labour Force Survey, 2008 (for UK, France, Belgium and Sweden); European Social Survey (2002–2010 for Europe).

	UK			FRANCE			BELGIUM			SWEDEN			EUROPE		
	UNEM	OUT	PTM	UNEM	OUT	PTM	UNEM	OUT	PTM	UNEM	OUT	PTM	UNEM	OUT	PTM
Native	3.6	15	49.4	4.6	20.6	45.4	3.2	21.1	45.4	2.2	9.2	45.4	6.5	16.7	51.4
FE	2.6	9.3	47.4	7.1	25	34.3	4.6	21.5	50.3	4.6	7.4	43.7	10.4	16.4	36.3
SE	3.4	12.5	56.9	5.6	20.6	40.4	7.4	20.3	39.2	5.3	9.0	46.7	9.2	17.4	47.0
FNE	7.1	17.7	46.3	11.5	19.3	33.2	19.1	19.8	38.9	14.2	12.4	32.5	11.4	14.5	32.8
SNE	7.3	14.1	52.6	14.1	16.1	40.3	25.5	28.7	35.0	10.7	28.6	20.0	17	13.4	49.9
N	26,963			16,270			7,500			20,468			31,245^1^		

Note

UNEM–percent unemployed, OUT- percent out of the labor force, PTM–percent engaged in professional, technician and managerial jobs (PTM) out of employed

FE–First Generation European, SE–Second Generation European, FNE–First Generation non-European, SNE–Second Generation non-European

1. Absolute number before weighting procedure.

**Table 2:** Immigrants in the labor market of West European countries: Descriptive statistics, women, %. Data Source: EU Labour Force Survey, 2008 (for UK, France, Belgium and Sweden); European Social Survey, 2002–2010 (for Europe).

	UK			FRANCE			BELGIUM			SWEDEN			EUROPE		
	UNEM	OUT	PTM	UNEM	OUT	PTM	UNEM	OUT	PTM	UNEM	OUT	PTM	UNEM	OUT	PTM
Native	2.4	27.9	42.1	5.3	28.6	45.4	3.1	34	45.0	2.2	12.7	48.9	6.3	27.9	49.4
FE	3	25.9	46.0	3.5	33.9	32.4	5.5	41.4	42.9	4.9	10.8	48.3	8.8	33.1	43.6
SE	2.9	25.3	48.6	5.3	27.7	39.6	12	31.3	39.0	3.2	11.6	45.8	8.1	26.3	48.4
FNE	3.9	46.9	43.3	10.3	45.4	29.7	10.3	53	31.8	10.9	24	28.7	12	39.5	32.6
SNE	5.2	29.5	49.6	11.7	31.3	41.3	19	40	37.5	15.6	18.8	33.3	13.6	28.3	51.9
N	30,071			17,423			7,448			20,628			33,066^1^		

Note: UNEM–percent unemployed, OUT- percent out of the labor force, PTM–percent engaged in professional, technician and managerial jobs (PTM) out of employed

FE–First Generation European, SE–Second Generation European, FNE–First Generation non-European, SNE–Second Generation non-European

1. Absolute number before weighting procedure.

At the same time, the data demonstrates that in all four countries, the proportion of unemployed among non-European immigrants (first- and second-generation) is substantially higher than that of European-origin immigrants. Moreover, in France and Belgium the differences in the percentage of unemployed (i.e. those who experience difficulty in finding employment) by region of origin are much more pronounced among men in the second-generation (i.e. sons of immigrants) than among first-generation immigrant men (14.1% and 5.6% for non-European and European immigrants respectively, in France; 25.5% and 7.4% for non-European and European immigrants respectively, in Belgium). It is important to emphasize that in all countries, second-generation non-European men experience greater disadvantage in finding employment not only in comparison to native-born without a migrant background or second-generation Europeans, but also in comparison to first-generation immigrants of European origin.

Among women, the percentage of unemployed among European-origin immigrants (first- and second-generation) is quite similar to that among native-born (with the exception of second-generation in Belgium). In all four countries, there is a higher proportion of unemployed among non-European immigrants than among the native-born population and immigrants of European origin. It is interesting to note that the percentage of women who are out of the labor force is substantially lower among second-generation women of non-European origin than among non-European first-generation immigrants. However, the proportion of unemployed second-generation non-European women is higher than that for first-generation non-European immigrant women.

Tables 1 and 2 also display the percentage of native born and immigrants employed in high-status lucrative occupations (professional, managerial and technicians) out of the overall employed population. While the descriptive data reveal that immigrants, especially those of non-European origin, are at a disadvantage with regards to finding employment, the 'picture' becomes quite different and somewhat more complex when the analysis focuses only on the economically active labor force. In the UK (representing a liberal-welfare state regime and flexible labor market) the percentage of first-generation immigrants who have attained high-status jobs is quite similar to that of the native-born population, while the percentage of second-generation immigrants employed in high-status occupations is even higher than that detected among native-born population (regardless of the immigrants' gender and region of origin). In France, first-generation immigrants, regardless of origin and gender, are underrepresented in high-status occupations in comparison to both second-generation and the native-born population. In Belgium and Sweden, the proportion of non-European immigrants employed in high-status occupations is lower than that of immigrants of European origin.

The data (see S2 Appendix) also reveal differences in socio-demographic attributes across sub-populations, such as age and education. For example, second-generation non-European immigrants are the youngest sub-group in all four countries. First-generation European immigrants are older than the native-born population in France and Sweden, but are younger than the native-born population in the UK. With regards to education, in France, Sweden and the UK, first-generation immigrant women of non-European origin are characterized by a lower level of formal education than that of native women; however, the educational level of second-generation women of non-European origin is somewhat higher than that of native-born women. Among men, the data varies substantially across countries. For example, in the UK non-European origin immigrants (first- and second-generation) and second-generation of European origin are characterized by a higher level of education than that of native-born men. In France, the level of education among immigrants of European origin (first- and second-generation) is somewhat lower than that of the native-born population, but the level of education of non-European origin immigrants is quite similar to that of native-borns. In Belgium, first-generation immigrants, whether of European or non-European origin, possess a bit higher level of education, while second-generation immigrants have a lower level of education than that of native-born men.

Differences in labor market position, as well as differential odds for achieving high-status jobs between sub-groups of immigrants and the native-born population, may also reflect differences in human capital and socio-demographic attributes such as education and age. Thus, in the following sub-sections, we report the effect of region of origin and generation on labor market outcomes, net of variations in socio-demographic attributes.

### Predicting modes of employment

The findings presented in Tables 3 and 4 demonstrate that in all four countries, the relative odds of being either 'unemployed' or 'out of the labor force' tend to decrease with level of education, and to be lower among married persons. The relationship between age and odds of being unemployed (as well as 'out of the labor force') are negative, taking a curve-linear form (as implied by positive and statistically significant coefficients of age squared).

**Table 3:** Immigrants in the labor market of West European countries: Exponents of coefficients [95% confidence interval] from multinomial regressions predicting odds for being unemployed/out of the labor force (versus employed), men. Data Source: EU Labour Force Survey, 2008 (for UK, France, Belgium and Sweden); European Social Survey, 2002–2010 (for Europe).

	UK^1^		FRANCE^1^		BELGIUM^1^		SWEDEN^1^		EUROPE^2^	
	UNEM	OUT	UNEM	OUT	UNEM	OUT	UNEM	OUT	UNEM	OUT
High Education^3^	.57* [.5, .7]	.78* [.7, .9]	.68* [.6, .8]	.64* [.6, .7]	.56* [.4, .7]	.49* [.4, .6]	.94 [.7, 1.2]	.70* [.6, .8]	—	—
Low Education^3^	1.82* [1.6, 2.1]	2.29* [2.1, 2.5]	1.91* [1.6, 2.2]	1.72* [1.5, 1.9]	1.82* [1.4, 2.3]	1.90* [1.6, 2.2]	1.92* [1.6, 2.3]	1.91* [1.7, 2.1]	—	—
Education in years	—	—	—	—	—	—	—	—	.91* [.89, .92]	.96* [.94, .96]
Married	.31* [.3, .4]	.40* [.3, .4]	.41* [.3, .5]	.60* [.5, .7]	.35* [.3, .4]	.46* [.4, .5]	.54* [.4, .7]	.54* [.5, .6]	.31* [.3, .35]	.51* [.5, .55]
Age	.89* [.8, .9]	.74* [.7, .75]	.85* [.8, .9]	.47* [.46, .48]	.92* [.8, 1.0]	.53* [.5, .55]	.79* [.7, .8]	.67* [.6, .7]	.87* [.8, .9]	.56* [.54, .56]
Age Square	1.001* [1.001, 1.002]	1.004* [1.004, 1.004]	1.002* [1.001, 1.002]	1.01* [1.009, 1.01]	1.001* [1.000, 1.002]	1.008* [1.008, 1.009]	1.003* [1.002, 1.003]	1.005* [1.005, 1.005]	1.002* [1.001, 1.002]	1.007* [1.007, 1.008]
First generation Europe^4^	.62* [0.4, 0.9]	.76* [.6, .9]	2.06* [1.4, 2.9]	.90 [.7, 1.2]	1.64* [1.0, 2.6]	.91 [.7, 1.2]	2.38* [1.6, 3.4]	.83 [.6, 1.1]	1.91* [1.6, 2.3]	1.40* [1.2, 1.6]
Second generation Europe^4^	1.12 [0.6, 2.1]	1.10 [.7, 1.6]	1.17 [.7, 1.9]	1.11 [.8, 1.5]	2.31* [1.2, 4.4]	1.51 [.9, 2.4]	2.62* [1.6, 4.2]	1.49* [1.03, 2.2]	1.43* [1.1, 1.9]	1.19 [.9, 1.5]
First generation non Europe^4^	2.82* [2.3, 3.5]	2.24* [1.9, 2.6]	3.26* [2.6, 4.0]	1.10 [.9, 1.3]	9.21* [6.7, 12.6]	2.28* [1.6, 3.1]	8.85* [6.9, 11.3]	2.47* [1.9, 3.2]	2.23* [1.9, 2.6]	1.54* [1.3, 1.8]
Second generation non European^4^	1.78* [1.2, 2.6]	1.60* [1.2, 2.1]	2.54* [1.9, 3.4]	1.45* [1.1, 1.9]	9.09* [5.2, 15.7]	2.54* [1.5, 4.4]	4.41* [1.2, 15.6]	4.35* [1.8, 10.6]	2.41* [1.9, 3.1]	.91 [.7, 1.2]
Nagelkerke–Pseudo R-Square	.198		.397		.394		.162		.285	

1. Model also includes very small categories ‘first generation other Europe’ and ‘second generation other Europe’ for control purposes (coefficient are presented in S4 Appendix).

2. Model includes also a series of country dummy variables and round dummy variables:

Exponents of coefficients for "Unemployed" are: ESS2 = 1.16*, ESS3 = 1.04. ESS4 = 0.95, ESS5 = 1.01, Switzerland = 0.41*, Germany = 1.55*, Denmark = 0.75, France = 1.07, UK = 1.12, Netherlands = 0.66*, Norway = 0.59*, Sweden = 0.59*

Exponents of coefficients for "Out of the labor force" are: ESS2 = 1.07, ESS3 = 0.89*. ESS4 = 0.84*, ESS5 = 0.89*, Switzerland = 0.43, Germany = 0.82*, Denmark = 0.64*, France = 1.02, UK = 1.12*, Netherlands = 0.75*, Norway = 0.47*, Sweden = 0.39*

3. Middle Level of Education is comparison category

4. Native population is comparison category

*p<0.05

**Table 4:** Immigrants in the labor market of West European countries: Exponents of coefficients [95% confidence interval] from multinomial regressions predicting odds for being unemployed/out of the labor force (versus employed), women. Data Sources: EU Labour Force Survey, 2008 (for UK, France, Belgium and Sweden); European Social Survey, 2002–2010 (for Europe).

	UK^1^		FRANCE^1^		BELGIUM^1^		SWEDEN^1^		EUROPE^2^	
	UNEM	OUT	UNEM	OUT	UNEM	OUT	UNEM	OUT	UNEM	OUT
High Education^3^	.47* [.4, .6]	.58* [.5, .6]	.56* [.4, .7]	.57* [.5, .6]	.40* [.3, .5]	.40* [.3, .5]	.49* [.4, .6]	.56* [.5, .6]	—	—
Low Education^3^	1.66* [1.4, 1.9]	2.45* [2.3, 2.6]	1.92* [1.6, 2.2]	1.96* [1.8, 2.1]	2.02* [1.5, 2.7]	2.06* [1.8, 2.4]	1.84* [1.5, 2.2]	2.14* [1.9, 2.4]	—	—
Education in years	—	—	—	—	—	—	—	—	.91* [.9, .91]	.94* [.93, .95]
Married	.40* [.3, .5]	.99 [.9, 1.05]	.71* [.6, .8]	1.58* [1.4. 1.7]	.54* [.4, .7]	1.28* [1.1, 1.4]	.81* [.7, 1.0]	.86* [.8, .9]	.50* [.46, .54]	1.44* [1.4, 1.5]
Age	.94* [.9, 1.0]	.75* [.74, .76]	.91* [.8, .9]	.58* [.56, .6]	.98 [.9, 1.0]	.61* [.58, .62]	.81* [.76, .84]	.71* [.69, .72]	.90* [.88, .92]	.67* [.65, .67]
Age Square	1.000 [1.000, 1.001]	1.004* [1.003, 1.004]	1.001* [1.000, 1.001]	1.007* [1.007, 1.007]	1.00 [0.999, 1.001]	1.006* [1.006, 1.007]	1.002* [1.002, 1.003]	1.004* [1.004, 1.005]	1.001* [1.001, 1.001]	1.005* [1.005, 1.005]
First generation Europe^4^	1.11 [.8, 1.6]	1.13 [.97, 1.3]	.80 [.5, 1.3]	.98 [.8, 1.2]	2.15* [1.4, 3.2]	1.39* [1.1, 1.7]	2.60* [1.8, 3.6]	.85 [.7, 1.1]	1.70* [1.4, 2.0]	1.40* [1.3, 1.6]
Second generation Europe^4^	1.39 [.7, 2.6]	1.12 [.9, 1.4]	.97 [.6, 1.5]	1.16 [.9, 1.5]	3.50* [2.0, 6.0]	1.32 [.9, 1.9]	1.48 [.8, 2.7]	1.29 [.9, 1.8]	1.19 [.9, 1.6]	0.99 [.8, 1.2]
First generation non Europe^4^	2.47* [1.9, 3.2]	3.20* [2.9, 3.5]	2.71* [2.2, 3.3]	2.69* [2.3, 3.1]	5.06* [3.4, 7.4]	3.89* [3.0, 4.9]	6.30* [4.8, 8.2]	3.30* [2.7, 3.9]	2.66* [2.2, 3.2]	2.24* [2.0, 2.5]
Second generation non European^4^	1.94* [1.3, 2.6]	1.74* [1.4, 2.1]	1.97* [1.5, 2.6]	1.92* [1.5, 2.4]	6.46* [3.6,11.7]	2.48* [1.5, 4.0]	5.17* [1.9, 14.0]	1.79 [.7, 4.5]	2.26* [1.7, 2.9]	1.09 [.9, 1.3]
Nagelkerke–Pseudo R-Square	.203		.300		.375		.164		.375	

1. Model also includes very small categories ‘first generation other Europe’ and ‘second generation other Europe’ for control purposes (coefficient presented in S4 Appendix).

2. Model includes also a series of country dummy variables and round dummy variables:

Exponents of coefficients for "Unemployed" are: ESS2 = 1.1, ESS3 = 0.96. ESS4 = 0.76*, ESS5 = 0.82*, Switzerland = 0.2*, Germany = 0.86, Denmark = 0.47*, France = 0.75*, UK = 0.4*, Netherlands = 0.37*, Norway = 0.3*, Sweden = 0.43*

Exponents of coefficients for "Out of the labor force" are: ESS2 = 0.89*, ESS3 = 0.82*. ESS4 = 0.77*, ESS5 = 0.77*, Switzerland = 0.74, Germany = 1.03, Denmark = 0.62*, France = 0.95, UK = 1.04, Netherlands = 1.00, Norway = 0.5*, Sweden = 0.44*

3. Middle Level of Education is comparison category

4. Native population is comparison category

*p<0.05

The results presented in Table 3 demonstrate that among men–except for immigrants of European origin in the UK–first-generation immigrants, regardless of region of origin, have higher odds of being unemployed than the comparable native-born populations. The odds of second-generation European men being unemployed in the UK and France are not different from those of native men, as implied by the statically insignificant and small coefficients (representing net differences in odds between a sub-group of immigrants and European natives). However, the odds of unemployment among second-generation non-European men in the UK and France are higher than those among native-born European men, as made evident by the statistically significant coefficients (*Exp(b)* = 1.78 and *Exp (b)* = 2.54). In Belgium and Sweden, the odds of being unemployed among second-generation European men are higher than those of the native-born population, and even more so among non-European immigrants (*Exp(b)* = 9.09 in Belgium and *Exp(b)* = 4.41 in Sweden).

Among women, the relative net odds of unemployment among first-generation European immigrants are not different from those of native-born women in UK and in France, but higher than those of native-born women in Belgium and Sweden. There are no statistically significant differences in the odds of unemployment between second-generation Europeans and native-born women without migrant background in the UK, France and Sweden. In Belgium however, the odds of being unemployed among second-generation women of European origin are considerably higher than those of native-born women *(Exp(b) = 3*.*50)*, but even more so among first- and second-generation non-European women (*Exp(b)* = 5.06 and *Exp(b)* = 6.46, respectively). In general, in all four countries, the odds of non-European women being unemployed, regardless of generation, are substantially higher than those of native women. The disparity is especially extreme in Belgium. The results also reveal that the relative odds of immigrant women of non-European origin being out of the labor force are lower in the second-generation than in the first-generation.

The findings regarding unemployment (controlling for non-participation in the labor market) among second-generation women, thus, are in line with expectations derived from the segmented assimilation theory in the context of all countries, except Belgium (even though second-generation European immigrants also experience substantial disadvantages in comparison to Belgium native-born population, the employment disadvantage of second-generation non-European immigrants is especially high). That is, the relatively high odds of unemployment that are apparent among most sub-groups of first-generation immigrants are also apparent among second-generation of non-European origin. By way of contrast, second-generation women of European origin achieve parity with the native-born population. Among men, however, second-generation of European origin achieve parity (equal odds) with native-born only in the UK and France. In Belgium and Sweden, their odds for unemployment are higher than those of native-born; yet, their odds for unemployment are much lower than those of immigrants of non-European origin.

Generally speaking, the findings of the country-specific analysis reveal that despite differences in welfare state regimes and migration integration policies, and despite cross-country differences in the size of the disadvantage that immigrant sub-groups experience, the patterns of labor market incorporation among immigrants are quite similar across the four countries.

Because the comparisons based on a four-country analysis can be limited and may not be generalizable to other European countries, in order to re-affirm our findings, we present in furthermost right-hand of Tables 3 and 4 the results of the supplementary analysis of the pooled data for *nine* '*old immigration*' *Western European countries*. The findings resemble the results found in all four countries. Specifically, the odds of unemployment among non-European immigrant men are higher than those among European immigrant men, especially among the second-generation. For women, the relative odds of unemployment among first-generation European and non-European immigrants are higher than those of native-born women. At the same time, the odds of unemployment among second-generation women of European origin do not differ significantly (at conventional statistical standards) from those of native-born women; the size of coefficients is also rather small. By contrast, the odds of unemployment for second-generation non-European women are considerably higher than those for native-born women.

### Predicting the odds of employment in high-status occupations

The logistic regressions (presented in Tables 5 and 6) suggest that the odds of being employed in a high-status occupation (versus other occupations) tend to rise with level of education regardless of gender, and are likely to be higher among married men. Number of children, does not affect the relative odds among men in all countries. Among women, however, number of children tends to reduce the relative odds for attainment of high status jobs in the UK and in France.

**Table 5:** Immigrants in the labor market of West European countries: Exponents of coefficients [95% confidence interval] from logistic regressions predicting odds for being employed in PTM (professional, technician and managerial) occupations (versus being employed in other occupations), men. Data Source EU Labour Force Survey, 2008 (for UK, France, Belgium and Sweden); European Social Survey, 2002–2010 (for Europe).

	UK^1^	FRANCE^1^	BELGIUM^1^	SWEDEN^1^	EUROPE^2^
High Education^3^	8.10* [7.5, 8.7]	13.83* [12.3, 15.5]	8.90* [7.7, 10.3]	12.43* [11.3, 13.7]	—
Low Education^3^	.57* [.5, .6]	.51* [.4, .6]	.43* [.4, .5]	.32* [.3, .4]	—
Education in years	—	—	—	—	1.37* [1.35, 1.38]
Married	1.51* [1.4, 1.6]	1.19* [1.1, 1.3]	1.19* [1.0, 1.3]	1.56* [1.4, 1.7]	1.24* [1.1, 1.3]
Age	1.15* [1.1, 1.2]	1.06* [1.0, 1.09]	.98 [0.9, 1.0]	1.13* [1.1, 1.15]	1.09* [1.0, 1.1]
Age Square	.998* [.998, 0.999]	1.000 [0.999, 1.00]	1.000 [1.000, 1.001]	.999* [.999, .999]	.999* [.999, .999]
Number of Children	97 [.9, 1.0]	.96 [.9, 1.0]	1.04 [1.0, 1.1]	---	1.02 [.99, 1.05]
First generation European^4^	1.06 [.9, 1.3]	.72* [.5, .9]	1.25 [.9, 1.6]	.67* [.5, .8]	.54* [.5, .6]
Second generation European^4^	1.05 [.8, 1.4]	1.00 [.7, 1.3]	.89 [.5, 1.4]	1.10 [.8, 1.4]	.91 [.7, 1.1]
First generation non European^4^	.69* [.6, .8]	.51* [.4, .6]	.68* [.5, .9]	.32* [.2, .4]	.41* [.3, .5]
Second generation non European^4^	.91 [.7, 1.1]	0.92 [.7, 1.2]	1.29 [.6, 2.7]	1.18 [.3, 4.4]	1.06 [.8, 1.3]
Nagelkerke–Pseudo R-Square	.305	.375	.352	.368	.289

1 Model also includes very small categories ‘first generation other Europe’ and ‘second generation other Europe’ (coefficient are presented in S6 Appendix).

2. Model includes also a series of country dummy variables and round dummy variables:

Exponents of coefficients are: ESS2 = 1.3*, ESS3 = 0.93. ESS4 = 1.09, ESS5 = 1.03, Switzerland = 1.9*, Germany = 0.57, Denmark = 0.74*, France = 1.07, UK = 0.71*, Netherlands = 1.11, Norway = 0.73*, Sweden = 1.03

3. Middle Level of Education is comparison category

4. Native population is comparison category

*p<0.05

**Table 6:** Immigrants in the labor market of West European countries: Exponents of coefficients [95% confidence interval] from logistic regressions predicting odds for being employed in PTM (professional, technician and managerial) occupations (versus being employed in other occupations), women. Data Source EU Labour Force Survey, 2008 (for UK, France, Belgium and Sweden); European Social Survey, 2002–2010 (for Europe).

	UK^1^	FRANCE^1^	BELGIUM^1^	SWEDEN^1^	EUROPE^2^
High Education^3^	7.48* [6.9, 8.0]	12.89* [11.5, 14.4]	10.33* [8.9, 12.0]	19.45* [17.8, 21.3]	—
Low Education^3^	.48* [.4, .5]	.36* [.3, .4]	.34* [.3, .4]	.34* [.3, .4]	—
Education in years	—	—	—	—	1.38* [1.3, 1.4]
Married	1.04 [1.0, 1.1]	.86* [.8, .9]	.93 [.8, 1.1]	1.19* [1.1, 1.2]	.95 [.9, 1.0]
Age	1.14* [1.11, 1.16]	1.10* [1.0, 1.1]	1.00 [.9, 1.0]	1.19* [1.1, 1.2]	1.05* [1.03, 1.07]
Age Square	.998* [.998, .999]	.999* [.999, 1.000]	1.000 [1.000, 1.001]	.998* [.998, .999]	1.000* [.999, 1.000]
Number of Children	.84* [.8, .9]	.91* [.8, .97]	1.06 [1.0, 1.1]	---	1.01 [.99, 1.05]
First generation European^4^	1.13 [.9, 1.3]	.68* [.5, .9]	.85 [.6, 1.1]	.73* [.6, .9]	.73* [.6, .8]
Second generation European^4^	1.10 [.8, 1.5]	.77 [.5, 1.0]	1.18 [.7, 2.0]	.92 [.7, 1.2]	.91 [.7, 1.1]
First generation non European^4^	.90 [.8, 1.0]	.60* [.5, .8]	.65* [.4, 1.0]	.26* [.2, .3]	.41* [.3, .5]
Second generation non European^4^	.98 [.7, 1.2]	.87 [.6, 1.2]	1.05 [.5, 2.3]	.43 [.1, 1.3]	1.20 [.9, 1.5]
Nagelkerke–Pseudo R-Square	.320	0.420	.402	.496	.265

1. Model also includes very small categories ‘first generation other Europe’ and ‘second generation other Europe’ for control purposes (coefficient are presented in S6 Appendix).

2. Model includes also a series of country dummy variables and round dummy variables:

Exponents of coefficients are: ESS2 = 1.08, ESS3 = 0.89*. ESS4 = 0.87*, ESS5 = 1.02, Switzerland = 1.93*, Germany = 0.78*, Denmark = 0.92, France = 0.92, UK = 0.62*, Netherlands = 1.64*, Norway = 0.81, Sweden = 1.07

3. Middle Level of Education is comparison category

4. Native population is comparison category

*p<0.05

The results reveal that in all four countries, the odds of second-generation immigrants–both of European and of non-European origin–to be employed in high-status occupations do not differ from those of natives (as evident by the size and significance level of the coefficients). These results are similar for both men and women. That is, after taking into consideration differences in socio-demographic attributes (e.g. age, education, marital status, number of children) second-generation men and women seem to have quite similar odds of attaining high status jobs as the other native-born populations.

As for the first-generation immigrants, the net odds of non-European immigrant men attaining employment in high-status occupations are lower than those of the native-born population in all four countries. This finding is clearly evident by the statistically significant and substantial coefficients ((*Exp(b)* = 0.69 for UK; *Exp(b)* = 0.51 for France; *Exp(b)* = 0.68 for Belgium; *Exp(b)* = 0.32 for Sweden). The net odds of first-generation European immigrants being employed in high-status occupations are lower than those of native-born men in France and Sweden, but not different from those of the native-born population in UK and Belgium. Among women, regardless of region of origin, the relative odds of first-generation immigrants being employed in high-status occupations are lower than those for the native-born populations in France and Sweden. In the UK, first-generation immigrant women and the native-born population have similar odds for attainment of high status jobs.

In general, examination of the relative odds for attainment of high-status occupations provides support to the arguments derived from the classic assimilation model: labor market disadvantages (as reflected in the low odds of employment in high-status occupations) are most evident among first-generation immigrants, but vanish in the second-generation. This finding is observed for immigrants from both regions of origin. The results of the supplementary analysis for the *nine* '*old immigration*' *Western European countries* are highly similar to those observed in the four countries. Whereas the relative odds of first-generation immigrants–whether of European or non-European origin–for attainment of high-status occupation are substantially lower than those for natives, the odds for second-generation immigrants are quite similar to those for natives, regardless of region of origin.

## Discussion

The results of the study reveal that patterns of labor force incorporation vary considerably across origin groups and across generations, but not as much across countries. Whereas the likelihood of becoming economically active and finding employment is influenced by region of origin–not only among first-generation immigrants, but also among the offspring of immigrants–the likelihood of attaining a high-status job is influenced mostly by immigrant status. Regardless of origin and gender, once second-generation, whether men or women, join the economically active labor force, their odds of attaining high status jobs become similar to that of the native-born population without a migrant background. These findings underscore the importance of distinguishing between the two aspects of immigrants' incorporation into the labor markets of host societies.

Specifically, immigrant men of non-European origin are more likely to be unemployed than immigrants of European origin in all four countries (as well as in Western Europe as a combined category). Moreover, the relative employment disadvantage among immigrant men of non-European origin (as compared to European-origin immigrants) is especially pronounced in the second-generation. The findings for immigrant women demonstrate that the net odds of unemployment (as well as for not being economically active) are higher among non-European immigrants of both the first- and second-generations than among native-born European women in the UK, Sweden, France and Belgium (and in Western Europe as whole). By contrast, the odds of unemployment for second-generation women of European origin are similar to those of native-born European women without a migrant background (except for Belgium).

Taking into account that the results related to unemployment were obtained when controlling for different rates of non-participation in the active labor force across immigrant sub-groups, and when controlling for education and age, it may imply that a sizable proportion of non-European immigrants and their descendants, both men and women, face substantial difficulties in integrating into the labor market of Western European countries. European immigrants, especially in the second-generation, however, seem to experience less difficulty than non-Europeans in incorporating into the labor force. Apparently, the impact of region of origin on the likelihood of finding employment in European countries is in line with expectations derived from the 'segmented assimilation model' [26], [29]. According to this approach, immigrants of non-European origin and their offspring suffer from structural barriers including discrimination that prevent successful integration into the host society. They are constrained by ethnic hierarchies, which restrict their access to employment opportunities [29], [31].

The analysis also reveals that the relative odds of labor force participation among second-generation non-European women are significantly higher than those of first- generation immigrants. Yet, the odds of unemployment among second-generation non-European women are only slightly lower than those for first-generation immigrant women. Whereas the relatively low rates of labor force participation among non-European women could be driven, at least in part, by traditional gender roles and values of seclusion that dominate several non-European communities [34], [51], the data show that in the second-generation, more non-European women are joining the economically active labor force, but still experience difficulties in finding employment. The increased rate of labor force participation among second-generation non-European women may indicate some form of growing cultural assimilation. However, the high rates of unemployment among second-generation non-European women can be explained by constrains associated with occupational gender segregation. Women in general, and non-European women in particular, have to compete for a relatively small number of occupations and jobs (mostly semiprofessional, clerical, and service-related jobs) [52], [53].

Results related to the attainment of occupational status as a second aspect of labor market incorporation show that first-generation immigrants, regardless of origin (and regardless of gender) are less likely to attain high-status jobs in the four countries (and in Western Europe as whole). At the same time, the likelihood of second-generation, of both European and non-European origin, to be employed in high-status jobs (versus low-status jobs) are similar to those of the comparable native-born populations. The findings indicate that in the second-generation, the odds of attaining occupational positions in European countries are more dependent on the human-capital attributes of individuals than on their origin, as predicted by the 'classic assimilation model '[21], [24], [32]. That is, insufficient proficiency in the domestic language, limited access to information and social networks, low level of transferability of professional skills and education lead to disadvantage of first generation immigrants in attaining high status jobs. However, because most second generation have acquired fluency of the host country's language and domestic educational and professional credentials, they do not experience such disadvantages, regardless of their origin [54].

Possible explanation as to why not only non-European immigrants but also their offspring experience a substantial relative disadvantage in finding employment, but not in attaining high-status job, can be found in the argument that ethnic discrimination takes place mostly in the very early stages of the hiring process. Experimental studies on the topic showed that ethnic minorities in European countries (whether foreign born or second generation) have much lower chances to be considered for a job, receive callback for a job interview or to be contacted by an employer [55], [56], [57].

Note that relative labor market disadvantages in finding employment and in attaining high-status jobs seem to be more pronounced among first generation immigrants of non-European origin as compared to immigrants of European origin. These results are in line with the contention that immigrants belonging to ethnic minorities and those arriving from less-developed economies are more likely to face discrimination and are less able to successfully convert their skills and professional credentials to economic outcome. This is partially so because employers in host countries are likely to devalue educational and professional credentials of immigrants belonging to ethnic minorities and those arriving from less developed countries [58], [59], [60].

A comparison of the results across the four countries reveals that in Sweden, percentage of women who belong to 'out of the labor force' category is lower than the percentage in any other country, regardless of migrant status and regardless of origin. It would seem that the socio-democratic welfare state regime and 'family-friendly' policies of the country increase labor force participation of all women, including immigrant women. These results are in line with Kesler [11], who suggested that low joblessness among first-generation immigrant women in Sweden as compared to Germany and the UK, can be explained by institutional support for women's employment.

The disadvantages experienced by immigrants in finding employment seem to be more pronounced in Belgium than in the other countries. Belgium is the single country where the likelihood of being unemployed for all sub-groups–first and second-generation immigrants of both European and non-European origin regardless of gender–is higher than that of the native-born population. The relative disadvantage of first-generation immigrants in the attainment of high-status occupational positions seems to be least pronounced in the liberal economy of the UK and most pronounced in the socio-democratic welfare state of Sweden. Migrations channels, introduced in the mid-1990s in the UK for highly skilled immigrants on the one hand [45], and the increased flow of refugees to Sweden [43] on the other, may provide possible explanations for the differences between the two countries with regards to immigrants' odds of attaining high-status occupations.

Indeed, one cannot ignore the possible effects of specific country of origin, religious denomination and cultural values (over and above the very broad category, European vs non-European region of origin) on differential modes of labor market integration. Such factors might play a significant role in influencing the differential patterns of labor market incorporation of immigrants in countries of destination. As discussed earlier in the paper, immigrant populations arriving in the UK, Sweden, Belgium and France differ by country of origin and by their ethnic and religious composition. Nevertheless, and despite such differences, the data of the present analysis demonstrates–rather convincingly–common patterns across generations and across the broad two categories of region of origin in the four countries and in Western Europe as a whole.

Indeed, country-specific findings reported here were limited to only four West European countries. Nevertheless and despite this limitation, the findings are consistent with those obtained from the nine-nation pooled data analysis, strengthening our confidence in the results and conclusions. Likewise, the small number of sampled cases of immigrants (especially in the second generation) prevented us from conducting more detailed analysis regarding region of origin, education and occupational categories. It is our hope that our analysis will stimulate future research on this important topic when more detailed data become available.

## Conclusions

This article focuses on two aspects of labor market incorporation (i.e. labor force status and attainment of occupational status) of first- and second-generation immigrants from European and non-European regions of origin in the UK, France, Sweden and Belgium (as well as in a combined category of nine Western European countries). The results presented here imply that non-European origin is associated with greater disadvantage in finding employment, while immigrant status (i.e. generation) is related to disadvantages in achieving high-status occupations. The disadvantages faced by immigrants of non-European origin, even in second-generation, in all four countries in finding employment may have significant consequences for future ethnic relations and social solidarity in Europe. Therefore, the findings presented by this article and the issues addressed here warrant further investigation, not only by social scientists but also by policy makers.

## Supporting information

S1 Appendix Sample size by immigration status. (DOC)

S2 Appendix Age (mean values) and education (% of high education). (DOC)

S3 Appendix Coefficients of linear probability model predicting probability for being unemployed. (DOC)

S4 Appendix Exponents of coefficients for 'other Europe' category from multinomial regressions presented in Tables 3 and 4 predicting odds for being unemployed/out of the labor force (versus employed). (DOC)

S5 Appendix Exponents of coefficients from multinomial regressions predicting odds for being employed in PTM occupations/not employed (versus being employed in other occupations). (DOC)

S6 Appendix Exponents of coefficients for 'other Europe' category from logistic regressions presented in Tables 5 and 6 predicting odds for being employed in PTM occupations (versus being employed in other occupations). (DOC)

S7 Appendix Coefficients of Linear Probability Model predicting probability for being employed in PTM occupations, employed (DOC)
