# Supplementary material for: Labor force participation, unemployment and occupational attainment among immigrants in West European countries
Source: PLoS One. 2017 May 5;12(5):e0176856. doi: 10.1371/journal.pone.0176856 (PMC5419508; doi:10.1371/journal.pone.0176856)
Supplement: S1 Appendix — (DOC) [file pone.0176856.s001.doc]

**S1 Appendix.** Sample size by immigration status

|  | UK | FRANCE | BELGIUM | SWEDEN | EUROPE^1^ |
| --- | --- | --- | --- | --- | --- |
| MEN |  |  |  |  |  |
| Native | 23614 | 13806 | 6414 | 18636 | 27656 |
| First generation European | 940 | 505 | 456 | 734 | 1571 |
| Second generation European | 297 | 360 | 148 | 378 | 504 |
| First generation non European | 1658 | 1146 | 388 | 714 | 1233 |
| Second generation non European | 454 | 453 | 94 | 28 | 281 |
| WOMEN | | | | | |
| Native | 26178 | 14688 | 6429 | 18519 | 29086 |
| First generation European | 1124 | 564 | 532 | 867 | 1886 |
| Second generation European | 348 | 393 | 150 | 378 | 537 |
| First generation non European | 1882 | 1273 | 417 | 836 | 1243 |
| Second generation non European | 539 | 505 | 100 | 32 | 314 |

1. Absolute number before weighting procedure.
