# Supplementary material for: Labor force participation, unemployment and occupational attainment among immigrants in West European countries
Source: PLoS One. 2017 May 5;12(5):e0176856. doi: 10.1371/journal.pone.0176856 (PMC5419508; doi:10.1371/journal.pone.0176856)
Supplement: S2 Appendix — (DOC) [file pone.0176856.s002.doc]

**S2a Appendix.** Age (mean values) and Education (% of high education), MEN

|  | UK | | FRANCE | | BELGIUM | | SWEDEN | |
| --- | --- | --- | --- | --- | --- | --- | --- | --- |
|  | Age | High  Education | Age | High  Education | Age | High  Education | Age | High  Education |
| Native | 44 | 30 | 42 | 25 | 43 | 29 | 42 | 24 |
| FE | 38 | 26 | 48 | 18 | 44 | 32 | 46 | 33 |
| SE | 45 | 38 | 41 | 22 | 41 | 20 | 38 | 23 |
| FNE | 40 | 33 | 44 | 25 | 41 | 30 | 38 | 33 |
| SNE | 33 | 41 | 33 | 24 | 27 | 14 | 25 | 8 |

Note: FE – First Generation European, SE – Second Generation European, FNE – First Generation non-European,

SNE – Second Generation non-European

**S2b Appendix.** Age (mean values) and Education (% of high education), WOMEN

|  | UK | | FRANCE | | BELGIUM | | SWEDEN | |
| --- | --- | --- | --- | --- | --- | --- | --- | --- |
|  | Age | High  Education | Age | High  Education | Age | High  Education | Age | High  Education |
| Native | 44 | 31 | 43 | 29 | 43 | 34 | 43 | 35 |
| FE | 38 | 32 | 47 | 24 | 44 | 31 | 47 | 40 |
| SE | 45 | 40 | 41 | 27 | 40 | 23 | 40 | 30 |
| FNE | 40 | 28 | 42 | 19 | 39 | 25 | 38 | 32 |
| SNE | 33 | 41 | 33 | 30 | 28 | 24 | 26 | 38 |

Note: FE – First Generation European, SE – Second Generation European, FNE – First Generation non-European,

SNE – Second Generation non-European
