# Supplementary material for: Labor force participation, unemployment and occupational attainment among immigrants in West European countries
Source: PLoS One. 2017 May 5;12(5):e0176856. doi: 10.1371/journal.pone.0176856 (PMC5419508; doi:10.1371/journal.pone.0176856)
Supplement: S3 Appendix — (DOC) [file pone.0176856.s003.doc]

**S3a Appendix.** Coefficients of Linear Probability Model predicting probability for being unemployed**,** MEN in the active labor force.

|  | UK | FRANCE | BELGIUM | SWEDEN | EUROPE^1^ |
| --- | --- | --- | --- | --- | --- |
| High Education^2^ | -0.018*  (0.003) | -0.019*  (0.005) | -0.019*  (0.007) | -0.002  (0.003) | -- |
| Low Education^2^ | 0.031*  (0.004) | 0.043*  (0.005) | 0.034*  (0.007) | 0.025*  (0.004) | -- |
| Education in years | -- | -- | -- | -- | -0.006*  (0.000) |
| Married | -0.045*  (0.003) | -0.046*  (0.005) | -0.049*  (0.007) | -0.014*  (0.004) | -0.082*  (0.004) |
| Age | -0.006  (0.001) | -0.012*  (0.002) | -0.005*  (0.002) | -0.008*  (0.001) | -0.012*  (0.001) |
| Age Square | 0.000*  (0.000) | 0.000*  (0.000) | 0.000*  (0.000) | 0.000*  (0.000) | 0.000*  (0.000) |
| First generation European^3^ | -0.018*  (0.007) | 0.044*  (0.013) | 0.020^&^  (0.012) | 0.029*  (0.007) | 0.053*  (0.008) |
| Second generation European^3^ | 0.007  (0.013) | 0.007  (0.015) | 0.049*  (0.021) | 0.035*  (0.009) | 0.028*  (0.012) |
| First generation non European^3^ | 0.053*  (0.006) | 0.093*  (0.009) | 0.200*  (0.013) | 0.140*  (0.007) | 0.067*  (0.007) |
| Second generation non European^3^ | 0.034*  (0.010) | 0.091*  (0.013) | 0.287*  (0.028) | 0.119*  (0.041) | 0.101*  (0.013) |
| First generation other European^3^ | 0.26  (0.022) | 0.075  (0.023)* | 0.084  (0.026)* | 0.14  (0.007)* |  |
| Second generation other European^3^ | -0.28  (-0.068) | 0.077  (0.06) | 0.15  (0.03)* | 0.119  (0.041)* |  |
| Intercept | 0.203*  (0.017) | 0.346*  (0.029) | 0.166*  (0.040) | 0.195*  (0.015) | 0.436*  (0.022) |

1. Model includes also a series of country dummy variables and round dummy variables. Coefficients are: ESS2=0.015*, ESS3= 0.005. ESS4=-0.002, ESS5=0.003, Switzerland= -0.051*, Germany=0.035*, Denmark= -0.013, France=0.004, UK=0.006, Netherlands=-0.021*, Norway= -0.03*, Sweden= -0.036*

2. Middle Level of Education is comparison category

3. Native population is comparison category

*p<0.05^&^p=0.109 (in the respective logistic model the significance level of this coefficients is 0.038)

**S3b Appendix.**  Coefficients of Linear Probability Model predicting probability for being unemployed, WOMEN in the active labor force.

|  | UK | FRANCE | BELGIUM | SWEDEN | EUROPE^1^ |
| --- | --- | --- | --- | --- | --- |
| High Education^2^ | -0.022*  (0.003) | -0.019*  (0.005) | -0.041*  (0.007) | -0.017*  (0.003) | -- |
| Low Education^2^ | 0.023*  (0.003) |  | 0.053*  (0.10) | 0.027*  (0.004) | -- |
| Education in years | -- | -- | -- | -- | -0.008*  (0.000) |
| Married | -0.030*  (0.003) | -0.021*  (0.005) | -0.031*  (0.007) | -0.004  (0.003) | -0.056*  (.004) |
| Age | -0.003*  (0.001) | -0.008*  (0.002) | 0.000  (0.000) | -0.008*  (0.001) | -0.011*  (0.001) |
| Age Square | 0.000  (0.000) | 0.000*  (0.000) | -0.000  (0.000) | 0.000*  (0.000) | 0.000*  (0.000) |
| First generation European^3^ | 0.001  (0.007) | -0.017  (0.014) | 0.046*  (0.014) | 0.032*  (0.007) | 0.050*  (0.008) |
| Second generation European^3^ | 0.011  (0.012) | -0.003  (0.016) | 0.110*  (0.024) | 0.012  (0.010) | 0.019  (0.13) |
| First generation non European^3^ | 0.041*  (0.006) | 0.106*  (0.011) | 0.236*  (0.031) | 0.113*  (0.007) | 0.114*  (0.009) |
| Second generation non European^3^ | 0.031*  (0.010) | 0.070*  (0.015) | 0.277*  (0.064) | 0.140*  (0.034) | 0.089*  (0.014) |
| First generation other European^3^ | 0.094  (0.026)* | 0.129  (0.032)* | 0.117  (0.031)* | 0.113  (0.007)* |  |
| Second generation other European^3^ | -0.02  (0.108) | -0.008  (0.048) | 0.28  (0.064)* | 0.140  (0.034)* |  |
| Intercept | 0.142*  (0.016) | 0.312*  (0.033) | 0.133*  (0.047) | 0.210*  (0.016) | 0.508*  (0.025) |

1. Model includes also a series of country dummy variables and round dummy variables. Coefficients are: ESS2=0.007, ESS3= -0.002. ESS4=-0.021*, ESS5=-0.016*, Switzerland= -0.12*, Germany=-0.02*, Denmark= -0.06*, France=-0.03*, UK=-0.08*, Netherlands=-0.08*, Norway= -0.09*, Sweden= -0.07*

2. Middle Level of Education is comparison category

3. Native population is comparison category

*p<0.05
