# Supplementary material for: Labor force participation, unemployment and occupational attainment among immigrants in West European countries
Source: PLoS One. 2017 May 5;12(5):e0176856. doi: 10.1371/journal.pone.0176856 (PMC5419508; doi:10.1371/journal.pone.0176856)
Supplement: S4 Appendix — (DOC) [file pone.0176856.s004.doc]

**S4 Appendix.** Exponents of coefficients for ‘other Europe including Turkey’ category from multinomial regressions presented in Tables 3 and 4

predicting odds for being **unemployed/out of the labor force (versus employed)** ^1^

|  |  | UK | | FRANCE | | BELGIUM | | SWEDEN | |
| --- | --- | --- | --- | --- | --- | --- | --- | --- | --- |
|  |  | UNEM | OUT | UNEM | OUT | UNEM | OUT | UNEM | OUT |
| Men | First generation Other Europe | 1.75 | *2.20 | *2.8 | 1.00 | *3.83 | *3.17 | *5.62 | 1.3 |
|  | Second generation Other Europe | .00* | .96 | 2.22 | 1.7 | *44.9 | 1.45 | *3.61 | 2.2 |
| Women | First generation Other Europe | *4.33 | *4.2 | *2.9 | *3.73 | *3.75 | *3.3 | *6.7 | *2.7 |
|  | Second generation Other Europe | *.00 | 3.4 | 0.83 | 1.07 | *6.53 | *3.36 | *4.55 | 1.34 |

1. This very small categories were introduced for control purposes.
