# Supplementary material for: Labor force participation, unemployment and occupational attainment among immigrants in West European countries
Source: PLoS One. 2017 May 5;12(5):e0176856. doi: 10.1371/journal.pone.0176856 (PMC5419508; doi:10.1371/journal.pone.0176856)
Supplement: S5 Appendix — (DOC) [file pone.0176856.s005.doc]

**S5a Appendix.** Exponents of coefficients from multinomial regressions predicting odds for being employed in **PTM occupations/not employed (versus being employed in other occupations),** MEN

|  | UK | | FRANCE | | BELGIUM | | SWEDEN | | EUROPE^1^ | |
| --- | --- | --- | --- | --- | --- | --- | --- | --- | --- | --- |
|  | PTM | NOTEMP | PTM | NOTEMP | PTM | NOTEMP | PTM | NOTEMP | PTM | NOTEMP |
| High Education | 7.945** | 2.272* | 14.033* | 2.935* | 8.880* | 1.748* | 12.304* | 3.308* |  |  |
| Low Education | .568* | 2.000* | .518* | 1.497* | .439* | 1.568* | .333* | 1.480* |  |  |
| Education in years |  |  |  |  |  |  |  |  | 1.337* | 1.087* |
| Married | 1.452* | .425* | 1.135* | .586* | 1.149 | .473* | 1.533* | .642* | 1.219* | .473* |
| Age | 1.168* | .799* | 1.085* | .555* | 1.013 | .581* | 1.139* | .711* | 1.112* | .667* |
| Age Square | .998* | 1.003* | .999* | 1.008* | 1.000 | 1.007* | .999* | 1.004* | .999* | 1.005* |
| Number of children | .976 | 1.035 | .966 | .948 | 1.034 | .951 | -- | -- | 1.031 | .982 |
| First generation Europe | 1.009 | .800 | .647* | .993 | 1.173 | 1.118 | .160* | .980 | .516* | 1.268* |
| Second generation Europe | 1.094 | 1.115 | .924 | 1.102 | .964 | 1.667* | 1.106 | 1.875* | .883 | 1.226 |
| First generation non Europe | .727* | 2.070* | .506* | 1.343* | .678* | 3.871* | .321* | 2.905* | .394* | 1.499* |
| Second generation non European | 1.033 | 1.584* | 1.006 | 1.988* | 1.148 | 4.503* | .909 | 4.720* | 1.024 | 1.482* |
| First generation other Europe | 0.73* | 2.07* | 0.506* | 1.34* | 0.68* | 3.87* | 0.32* | 2.9* |  |  |
| Second generation other Europe | 1.03 | 1.58* | 1.006 | 1.99* | 1.15 | 4.5* | 0.91 | 4.72* |  |  |
| Nagelkerke – Pseudo R-Square | 0.330 | | 0.464 | | 0.455 | | 0.360 | | 0.349 | |

1. Model includes also a series of country dummy variables and round dummy variables:

Exponents of coefficients for "PMT" are: ESS2=1.3*, ESS3= 0.96. ESS4=1.1*, ESS5=1.07, Switzerland= 1.75*, Germany=0.59*, Denmark= 0.76*, France=1.03, UK=0.7*, Netherlands=1.1, Norway= 0.75*, Sweden= 1.007

Exponents of coefficients for "Not employed" are: ESS2=1.23*, ESS3= 0.92. ESS4=0.89*, ESS5=0.93, Switzerland= 0.56*, Germany=0.82*, Denmark= 0.63*, France=1.05, UK=0.71*, Netherlands=0.7*, Norway= 0.46*, Sweden= 0.43*

*p<0.05

**S5b Appendix.** Exponents of coefficients from multinomial regressions predicting odds for being employed in **PTM occupations/not employed (versus being employed in other occupations),** WOMEN

|  | UK | | FRANCE | | BELGIUM | | SWEDEN | | EUROPE^1^ | |
| --- | --- | --- | --- | --- | --- | --- | --- | --- | --- | --- |
|  | PTM | NOTEMP | PTM | NOTEMP | PTM | NOTEMP | PTM | NOTEMP | PTM | NOTEMP |
| High Education | 7.411** | 1.601* | 12.511* | 2.212* | 10.063* | 1.219* | 19.281* | 2.830* |  |  |
| Low Education | .481* | 2.028* | .377* | 1.541* | .357* | 1.761* | .355* | 1.714* |  |  |
| Education in years |  |  |  |  |  |  |  |  | 1.301* | 1.065* |
| Married | .996 | .763* | .875* | 1.069 | .897 | 1.011 | 1.179* | .912 | .920* | .921* |
| Age | 1.138* | .737* | 1.122* | .595* | 1.041 | .617* | 1.201* | .756* | 1.064* | .673* |
| Age Square | .998* | 1.004* | .999* | 1.007* | 1.000 | 1.006* | .998* | 1.003* | .999* | 1.005* |
| Number of children | .849* | 1.715* | .916* | 1.560* | 1.034 | 1.311* | -- | -- | 1.043* | 1.584* |
| First generation Europe | 1.096 | 1.384* | .550* | .797* | .908 | 1.448* | .692* | .959 | .730* | 1.363* |
| Second generation Europe | 1.054 | 1.170 | .781 | 1.074 | 1.055 | 1.698* | .950 | 1.309 | .951 | 1.029 |
| First generation non Europe | .983 | 2.982* | .573* | 2.049* | .553* | 3.249* | .280* | 2.720* | .470* | 1.947* |
| Second generation non European | 1.036 | 1.514* | .853 | 1.698* | .838 | 2.751* | .646 | 1.986 | 1.095 | 1.293* |
| First generation other Europe | 0.98 | 2.98* | 0.57* | 2.05* | 0.55* | 3.25* | 0.28* | 2.72* | --- | --- |
| Second generation other Europe | 1.03 | 1.51* | 0.85 | 1.7* | 0.84 | 2.75* | 0.64 | 1.98 | --- | --- |
| Nagelkerke – Pseudo R-Square | .357 | | .431 | | .463 | | .447 | | .264 | |

1. Model includes also a series of country dummy variables and round dummy variables:

Exponents of coefficients for "PMT" are: ESS2=1.04, ESS3= 0.9*. ESS4=0.91*, ESS5=1.02, Switzerland= 1.48*, Germany=0.73*, Denmark= 0.9, France=0.82*, UK=0.57*, Netherlands=1.46*, Norway= 0.78*, Sweden= 0.97

Exponents of coefficients for "Not employed" are: ESS2=0.95, ESS3= 0.81*. ESS4=0.74*, ESS5=0.77*, Switzerland= 0.69*, Germany=0.87*, Denmark= 0.54*, France=0.79*, UK=0.62*, Netherlands=0.96, Norway= 0.36*, Sweden= 0.38*

*p<0.05
