# Supplementary material for: Labor force participation, unemployment and occupational attainment among immigrants in West European countries
Source: PLoS One. 2017 May 5;12(5):e0176856. doi: 10.1371/journal.pone.0176856 (PMC5419508; doi:10.1371/journal.pone.0176856)
Supplement: S6 Appendix — (DOC) [file pone.0176856.s006.doc]

**S6 Appendix**. Exponents of coefficients for ‘other Europe including Turkey’ category from logistic regressions presented in Tables 5 and 6

predicting odds for being employed in **PTM occupations (versus being employed in other occupations)** ^1^

|  |  | UK | FRANCE | BELGIUM | SWEDEN |
| --- | --- | --- | --- | --- | --- |
| Men | First generation Other Europe | .88 | *.42 | *.47 | *.14 |
|  | Second generation Other Europe | 1.66 | .46 | 1.43 | .88 |
| Women | First generation Other Europe | .89 | *.29 | *.08 | *.28 |
|  | Second generation Other Europe | 1.68 | .93 | .84 | 1.3 |

1. This very small categories were introduced for control purposes.

*p<0.05
