# Supplementary material for: Labor force participation, unemployment and occupational attainment among immigrants in West European countries
Source: PLoS One. 2017 May 5;12(5):e0176856. doi: 10.1371/journal.pone.0176856 (PMC5419508; doi:10.1371/journal.pone.0176856)
Supplement: S7 Appendix — (DOC) [file pone.0176856.s007.doc]

**S7a Appendix.** Coefficients of Linear Probability Model predicting probability for being employed in **PTM occupations**, employed MEN.

|  | UK | FRANCE | BELGIUM | SWEDEN | EUROPE^1^ |
| --- | --- | --- | --- | --- | --- |
| High Education^2^ | 0.450*  (0.007) | 0.541*  (0.009) | 0.481*  (0.013) | 0.502*  (0.007) | -- |
| Low Education^2^ | -0.114*  (0.008) | -0.121*  (0.010) | -0.149*  (0.015) | -0.198*  (0.009) | -- |
| Education in years | -- | -- | -- | -- | 0.056*  (0.001) |
| Married | 0.080*  (0.007) | 0.031*  (0.01) | 0.031*  (0.014) | 0.082*  (0.007) | 0.045*  (0.007) |
| Age | 0.027*  (0.002) | 0.009*  (0.003) | -0.003  (0.00) | 0.021*  (0.002) | 0.018*  (0.002) |
| Age Square | 0.000*  (0.000) | -0.000  (0.000) | 0.000  (0.000) | 0.000  (0.000) | 0.000  (0.000) |
| Number of Children | -0.006  (0.004) | -0.007  (0.005) | 0.007  (0.007) | --- |  |
| First generation European^3^ | 0.010  (0.017) | -0.058*  (0.023) | 0.038  (0.024) | -0.065*  (0.017) | -0.120*  (0.014) |
| Second generation European^3^ | 0.010  (0.029) | -0.001  (0.026) | -0.022  (0.042) | 0.018  (0.024) | -0.024  (0.021) |
| First generation non European^3^ | -0.069*  (0.013) | -0.107*  (0.016) | -0.063*  (0.029) | -0.164*  (0.017) | -0.160*  (0.013) |
| Second generation non European^3^ | -0.015  (0.024) | -0.014  (0.025) | 0.044  (0.067) | 0.025  (0.116) | 0.011  (0.023) |
| First generation other European^3^ | -0.25  (0.054) | -0.119  (0.043)* | -0.112  (0.054)* | -0.164  (0.019)* |  |
| Second generation other European^3^ | 0.088  (0.154) | -0.128  (0.131) | 0.063  (0.08) | 0.025  (0.116) |  |
| Intercept | -0.240*  (0.040) | 0.025  (0.055) | 0.264*  (0.082) | -0.175*  (0.038) | -0.728*  (0.040) |

1. Model includes also a series of country dummy variables and round dummy variables. Coefficients are: ESS2=0.05*, ESS3= -0.008. ESS4=0.02*, ESS5=0.009*, Switzerland= 0.1*, Germany=-0.12*, Denmark= -0.05*, France=0.004, UK=-0.07*, Netherlands=0.02, Norway= -0.05*, Sweden= 0.001

2. Middle Level of Education is comparison category

3. Native population is comparison category

*p<0.05

**S7b Appendix.** Coefficients of Linear Probability Model predicting probability for being employed in **PTM occupations**, employed WOMEN.

|  | UK | FRANCE | BELGIUM | SWEDEN | EUROPE^1^ |
| --- | --- | --- | --- | --- | --- |
| High Education^2^ | 0.460*  (0.007) | 0.544*  (0.01) | 0.521*  (0.13) | 0.588*  (0.006) | -- |
| Low Education^2^ | -0.122*  (0.008) | -0.164*  (0.010) | -0.140*  (0.018) | -0.164*  (0.009) | -- |
| Education in years | -- | -- | -- | -- | 0.059*  (0.001) |
| Married | 0.007  (0.007) | -0.024*  (0.009) | -0.011  (0.013) | 0.026*  (0.006) | -0.010  (0.006) |
| Age | 0.023*  (0.002) | 0.015*  (0.003) | -0.001  (0.005) | 0.025*  (0.002) | 0.011*  (0.002) |
| Age^2 | 0.000*  (0.000) | 0.000*  (0.000) | 0.000  (0.000) | 0.000*  (0.000) | 0.000*  (0.000) |
| Number of Children | -0.031*  (0.004) | -0.014*  (0.005) | 0.011  (0.124) | --- |  |
| First generation European^3^ | 0.024  (0.016) | -0.058*  (0.02) | -0.027  (0.025) | -0.044*  (0.015) | -0.057*  (0.014) |
| Second generation European^3^ | 0.018  (0.027) | -0.043  (0.027) | 0.027  (0.045) | -0.013  (0.022) | -0.018  (0.021) |
| First generation non European^3^ | -0.019  (0.015) | -0.072*  (0.018) | -0.063^&^  (0.034) | -0.172*  (0.017) | -0.148*  (0.016) |
| Second generation non European^3^ | -0.004  (0.023) | -0.021  (0.025) | 0.006  (0.065) | -0.120  (0.084) | 0.037  (0.025) |
| First generation other European^3^ | -0.018  (0.063) | -0.175  (0.055)* | -0.240  (0.060)* | -0.172  (0.017)* |  |
| Second generation other European^3^ | 0.087  (0.301) | -0.009  (0.076) | -0.029  (0.136) | -0.120  (0.184) |  |
| Intercept | -0.145*  (0.040) | -0.061  (0.056) | 0.202*  (0.87) | -0.304*  (0.036) | -0.547*  (0.044) |

1. Model includes also a series of country dummy variables and round dummy variables. Coefficients are: ESS2=0.01, ESS3= -0.02*. ESS4=-0.02*, ESS5=0.01, Switzerland= 0.1*, Germany=-0.06*, Denmark= -0.008, France=-0.02, UK=-0.1*, Netherlands=0.09*, Norway= -0.04, Sweden= 0.01

2. Middle Level of Education is comparison category

3. Native population is comparison category

*p<0.05 ^&^p=0.065 (in the respective logistic model the significance level of this coefficients is 0.048)
